# Evaluation of the molecular bacterial load assay for detecting viable *Mycobacterium tuberculosis* in cerebrospinal fluid before and during tuberculous meningitis treatment

**DOI:** 10.1016/j.tube.2021.102084

**Published:** 2021-05

**Authors:** Hoang Thanh Hai, Wilber Sabiiti, Do Dang Anh Thu, Nguyen Hoan Phu, Stephen H. Gillespie, Guy E. Thwaites, Nguyen Thuy Thuong Thuong

**Affiliations:** a Oxford University Clinical Research Unit, Ho Chi Minh City, Viet Nam; b Nuffield Department of Medicine, University of Oxford, United Kingdom; c School of Medicine, Division of Infection and Global Health, University of St Andrews, United Kingdom; d Hospital for Tropical Diseases, Ho Chi Minh City, Viet Nam

**Keywords:** Tuberculosis meningitis, Cerebrospinal fluid, *Mycobacterium tuberculosis*, Viable bacterial load, 16S rRNA

## Abstract

New tools to monitor treatment response and predict outcome from tuberculous meningitis (TBM) are urgently required. We retrospectively evaluated the 16S rRNA-based molecular bacterial load assay (MBLA) to quantify viable *Mycobacterium tuberculosis* in serial cerebrospinal fluid (CSF) from adults with TBM. 187 CSF samples were collected before and during the first two months of treatment from 99 adults TBM, comprising 56 definite, 43 probable or possible TBM, and 18 non-TBM and preserved at −80°C prior to MBLA. We compared MBLA against MGIT culture, GeneXpert MTB/RIF (Xpert) and Ziehl-Neelsen (ZN) smear. Before treatment, MBLA was positive in 34/99 (34.3%), significantly lower than MGIT 47/99 (47.5%), Xpert 51/99 (51.5%) and ZN smear 55/99 (55.5%). After one month of treatment, MBLA and MGIT were positive in 3/38 (7.9%) and 4/38 (10.5%), respectively, whereas Xpert and ZN smear remained positive in 19/38 (50.0%) and 18/38 (47.4%). In summary, MBLA was less likely to detect CSF bacteria before the start of treatment compared with MGIT culture, Xpert and ZN smear. MBLA and MGIT positivity fell during treatment because of detecting only viable bacteria, whereas Xpert and ZN smear remained positive for longer because of detecting both live and dead bacteria. Sample storage and processing may have reduced MBLA-detectable viable bacteria; and sampling earlier in treatment may yield more useful results. Prospective studies with CSF sampling after 1–2 weeks are warranted.

## 1 Introduction

Tuberculous meningitis (TBM) is the most severe form of tuberculosis (TB). Caused by dissemination of *Mycobacterium tuberculosis* (*Mtb*) to the brain, it results in death or neurological disability in half of all cases. Many clinical trials have been conducted to improve treatment outcomes in TBM, but optimal anti-tuberculosis therapy remains uncertain [[1], [2], [3]]. In pulmonary tuberculosis (PTB), monitoring *Mtb* bacillary load before and during treatment has been found to be useful for examining treatment response, evaluating therapy efficiency and predicting outcome [4]. Numbers of *Mtb* in cerebrospinal fluid (CSF) of TBM patients are normally much lower than in PTB sputum samples, which may make it challenging to assess viable bacterial load during treatment [5,6]. Using GeneXpert MTB/RIF (Xpert) among 692 adults with TBM, we showed previously that pre-treatment bacterial load correlated with neurological severity at presentation and with the appearance of new neurological events after starting treatment [6]. However, whether pre-treatment bacterial load and bacterial clearance affect early treatment response, and in turn treatment outcome, is still unknown due to the limitations of current methods for measuring viable *Mtb* in TBM. A new rapid assay is therefore needed for TBM patients, particularly during the early phases of treatment [7,8].

CSF *Mtb* bacterial load can be inferred from MGIT culture and the Xpert assay. However, culture is slow and sensitive to contamination, and the Xpert assay is a DNA-based method that does not accurately reflect the viable bacterial numbers [9,10]. The molecular bacterial load assay (MBLA) based on 16S rRNA is able to rapidly quantify viable bacteria, and has been evaluated in PTB patients before and in the early stages of anti-TB treatment [9,[11], [12], [13], [14]].

Since the MBLA works well in PTB, we aimed to test MBLA performance in TBM. We compared *Mtb* detection and quantification in CSF samples by the MBLA method with conventional methods: MGIT, Xpert and Ziehl-Neelsen (ZN) stain.

## 2 Materials and methods

### 2.1 Participants, treatment and follow-up

We conducted a retrospective study of 117 cases of meningitis, comprising 99 cases with clinical and laboratory evidence suggestive of TBM and 18 cases with evidence suggestive of non-TBM. 187 CSF samples were collected from all of these patients before and during the first two months of anti-TB treatment. Participants were recruited as part of two clinical trials (ISRCTN61649292 and NCT02237365) conducted in the Hospital for Tropical Diseases (HTD), Vietnam from 2011 to 2016. Written informed consent was obtained from each participant or from an accompanying relative. Protocols were approved by the Oxford Tropical Research Ethics Committee in the United Kingdom, the institutional review boards of HTD and the Ethical Committee of the Ministry of Health in Vietnam [2,3,15].

Participants received a diagnosis following the uniform case definition for TBM clinical research [16]. TBM patients all received standard anti-tuberculosis chemotherapy TBM-treatment for 9 months, with a 3-month intensive phase consisting of oral isoniazid (5 mg/kg/day), rifampicin (10 mg/kg/day), pyrazinamide (25 mg/kg/day), ethambutol (20 mg/kg/day) and intramuscular streptomycin (20 mg/kg/day), followed by a 6-month continuation phase consisting of only rifampicin and isoniazid. Adjunctive dexamethasone was given to all patients for the first 6–8 weeks of treatment. In ISRCTN61649292 trial, patients were randomized to receive intensified therapy with levofloxacin (20 mg/kg) combined an increased dosage of rifampicin (15 mg/kg/day) or to receive placebo for the first two months. In NCT02237365 trial, patients were randomised to receive one of three arms: aspirin 81 mg, aspirin 1000 mg or placebo daily for the first two months. According to clinical trial protocols, lumbar puncture, unless clinically contraindicated, was performed before the start of treatment and then repeated after one month and two months to assess treatment response. All pre-treatment CSF samples were used to evaluate *Mtb* detection by MBLA. However, for evaluation of bacterial clearance during treatment, we only used sequential CSF samples at one month and two months from definite TBM.

### 2.2 Sample processing and microbiological examination

Participants underwent routine diagnosis of TBM. An average volume of 3–5 ml of CSF was used for mycobacterial diagnostic tests. The CSF was centrifuged for 15 min at 3000g. After discarding supernatant, approximately 700 μl of deposit was used for the diagnostic tests: 100 μl for ZN smear, 200 μl for MGIT culture, 200 μl for Xpert and 200 μl for storage at −80°C and later MBLA testing.

ZN smear, MGIT culture and Xpert were performed as previously described [2,3,15]. For data analysis, ZN smear, MGIT and Xpert results were recorded as positive or negative. In addition, for Xpert, average cycle threshold (Ct) values of five probes (excluding any delayed values due to rifampicin resistance) were converted into bacterial load log_10_ colony forming units per milliliter (log_10_ CFU/ml). A standard curve of Xpert Ct values versus CFU/ml of *Mycobacterium bovis* (bacillus Calmette-Guérin; NCTC 5692) was generated for conversion as previously described [6]. A value of 0 CFU/ml and Ct 40 were assigned if the Xpert result was negative.

### 2.3 RNA extraction and RT-qPCR for MBLA

To stabilize RNA during extraction, 800 μl of GTC buffer (4 M guanidine thiocyanate, 0.1 M Tris-HCl, and 1% β-mercaptoethanol) was added to 200 μl of stored CSF. RNA was extracted following the protocol previously described [12]. Briefly, 4 log_10_ CFU/ml of internal control was added and total RNA was extracted through three major steps involving cell wall disruption, RNA separation and purification, and removal of contaminated DNA. Ct values of 16S rRNA were measured by RT-qPCR and converted to log_10_ CFU/ml using our optimizing standard curve. A Ct of 35 was defined as the cut-off to determine a negative result for MBLA [9,12].

### 2.4 Inhibition and lower limit of detection of MBLA

BCG culture in 7H9 broth at OD_600_ of 0.6 was counted for CFU, and then was stabilized and stored in GTC buffer. To generate inhibitor-free samples, after CFU count, stored BCG was spiked into GTC at final concentrations ranging from low (1 log_10_) to high (7 log_10_) CFU/ml. To mimic CSF samples or sputum samples, stored BCG was spiked in pooled CSF or pooled sputum from five non-TB patients who have *Mtb*-negative by both ZN smear and Xpert, to the same concentrations above. MBLA, including RNA extraction and RT-qPCR, was performed with four replicates and 16S rRNA Ct values were recorded. To determine the inhibition of MBLA in clinical samples, Ct values from CSF or sputum samples were compared to those of GTC samples. The lower limit of detection (LOD) of MBLA in CSF or sputum was defined as the lowest number of CFU/ml that yielded a Ct value below 32.

### 2.5 Data analysis and statistics

Statistical analyses were performed using R version 4.0.3 (R Development Core Team, Vienna, Austria) and Graphpad Prism version 6. To determine inhibition of MBLA in clinical samples, Ct values measured in CSF or sputum samples were compared to those of the GTC control using Paired *t*-test. McNemar's test was used to compare detection rates of MBLA with those of MGIT, Xpert and ZN smear.

## 3 Results

### 3.1 Characteristics of participants

187 stored CSF samples from 117 suspected TBM participants were used for MBLA. Applying the uniform case definition for TBM clinical research, 99/117 (84.6%) participants were identified having TBM, comprising 56 cases of definite TBM, 43 cases of probable or possible TBM, and 18/117 (15.4%) were identified as non-TBM (13 cases of bacterial meningitis, 3 of viral and 2 fungal meningitis). Pre-treatment samples were available from all participants. Samples after one month and two months of anti-tuberculosis treatment were available in 38/117 (32.5%) and 32/117 (27.4%) definite TBM patients respectively.

Baseline characteristics of participants are shown in Table 1. Overall, the participants’ median age was 34.0 years [IQR 29.0; 45.0] in TBM and 37.0 years [IQR 30.2; 52.0] in non-TBM. The majority of participants were male: 68/99 (68.7%) in TBM and 11/18 (61.1%) in non-TBM. There were 30/99 (30.3%) cases of HIV co-infection in those with TBM, but only 2/18 (11.1%) in those without TBM. Both TBM and non-TBM patients had raised numbers of total CSF leukocytes (144 [IQR 59; 306] and 263 [IQR 133; 444] x10^3^ cells/ml, respectively), in which about 70% were lymphocytes.

**Table 1:** Baseline clinical characteristics of 117 participants stratified by TBM infection.

Characteristics	Non TBM		TBM	
	N = 18		N = 99	
	**No.**	**Summary** **Statistic**	**No.**	**Summary** **Statistic**
**Diagnostic category**	…	…	99	
Definite TBM	…	…	99	56 (56.6%)
Possible/Probable TBM	…	…	99	43 (43.4%)
**Age** (year), (median, IQR)	18	37.0 [30.2; 52.0]	99	34.0 [29.0; 45.0]
**Male gender** (N, %)	18	11 (61.1%)	99	68 (68.7%)
**CSF volume** (ml)	16	3.50 [3.50; 4.00]	99	4.50 [4.00; 6.00]
**HIV infected** (N, %)	18	2 (11.1%)	99	30 (30.3%)
**Glassgow Coma Scale**	…	…	99	15.0 [13.0; 15.0]
**CSF parameter**	18		99	
CSF leukocytes (x10^3^ cells/ml)	18	263 [133; 444]	99	144 [59.5; 306]
Neutrophil (%)	18	28.5 [24.0; 50.8]	91	24.0 [7.00; 60.5]
Lymphocytes (%)	18	72.0 [62.0; 76.0]	91	76.0 [39.5; 93.0]
Protein level (g/L)	…	…	98	1.49 [1.04; 2.18]
Glucose (mmol/L)	…	…	98	1.71 [1.20; 2.29]
Lactate level (mmol/L)	…	…	96	4.70 [3.70; 6.42]

Data are number and % of participants or median value [interquartile range].

AbbreviationsTBM: tuberculosis meningitis; CSF: cerebrospinal fluid; HIV: human immunodeficiency virus.

Summary statistics was only calculated for cases without missing data for the corresponding variable. Diagnostic categories were assigned according to the uniform case definition [14].

### 3.2 *Mtb* detection before treatment

**Figure 1:**
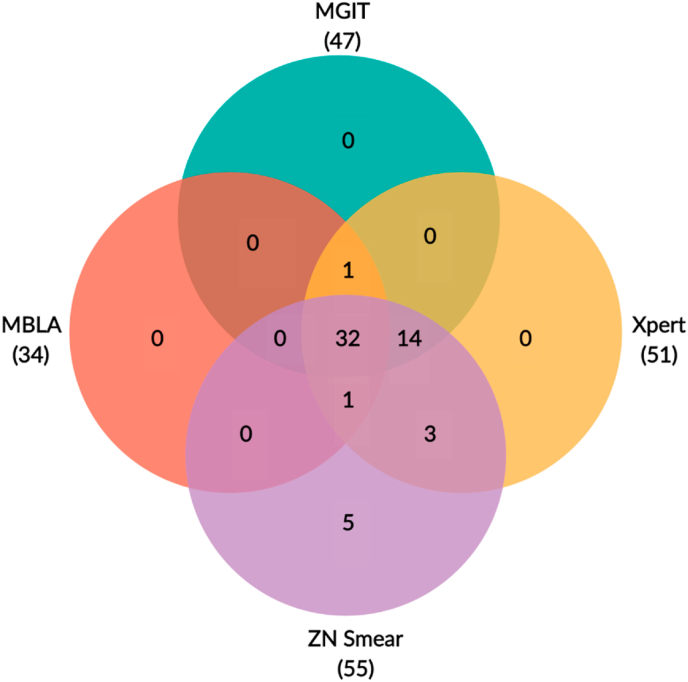
**Venn diagram of positive results of *Mtb* detection at baseline by MBLA, MGIT, Xpert and ZN Smear**. Results are for 99 TBM patients. Positive results for each test are in brackets.; Abbreviations: MBLA: molecular bacterial load assay; MGIT: mycobacterial culture by MGIT (Becton Dickinson, USA); Xpert: GeneXpert MTB/RIF (Cepheid); ZN smear: Ziehl–Neelsen smear.

The ability of MBLA to detect *Mtb* in 117 CSF samples collected before treatment was compared with that of MGIT culture, Xpert and ZN smear. In 99 TBM patients, 34/99 (34.3%) cases had *Mtb* detected in CSF by MBLA. In comparison, *Mtb* was detected in 47/99 (47.5%) by MGIT, 51/99 (51.5%) by Xpert, and 55/99 (55.6%) by ZN smear (Table 2). The detection rate of MBLA was significantly lower than that of MGIT (−13.2%; *P = 0.001*; McNemar's Test), Xpert (−17.2%; *P < 0.001*) and ZN smear (−21.3%; *P < 0.001*). Among the 34 TBM cases found positive by MBLA, 32 were positive by all other tests, 1 was Xpert positive at a low level and MGIT positive but ZN smear negative, and 1 was Xpert positive at a medium level and ZN smear positive but MGIT negative. No case was detected by MBLA alone (Fig. 1).

**Table 2:** *Mtb* detection rate by MBLA, MGIT, Xpert and ZN smear testing in CSF samples from all TBM, definite TBM patients defined by microbiological confirmation (definite TBM), TBM patients defined by MGIT culture positivity (MGIT+) and from non-TBM patients.

Methods	All TBM	*P*	Definite TBM	TBM by MGIT+	Non-TBM
	N = 99		N = 56	N = 47	N = 18
**MBLA**	34 (34.3%)	reference	34 (60.7%)	33 (70.2%)	0 (0.0%)
**MGIT**	47 (47.5%)	0.001	47 (83.9%)	47 (100%)	0 (0.0%)
**Xpert**	51 (51.5%)	<0.001	51 (91.1%)	47 (100%)	0 (0.0%)
**ZN smear**	55 (55.5%)	<0.001	51 (91.1%)	46 (97.9%)	0 (0.0%)

Data are number (%) of positive cases by methods.

*P* column shows *P* values of McNemar tests, which compares MBLA with each of remaining methods: MGIT culture, Xpert, ZN smear.; Abbreviations: TBM: tuberculosis meningitis; MBLA: molecular bacterial load assay; MGIT: mycobacterial culture by MGIT (Becton Dickinson, USA); Xpert: GeneXpert MTB/RIF (Cepheid); ZN smear: Ziehl–Neelsen smear.

The detection rate of MBLA in the 56 definite TBM cases was 34/56 (60.7%) and in the 47 TBM cases defined by MGIT culture positivity was 33/47 (70.2%) (Table 2). Although MBLA increased *Mtb* detection in microbiological confirmed subsets, the detection rates of MBLA were consistently lower than other routine tests. In the 18/117 patients diagnosed with non-TBM, *Mtb* was not detected by MBLA or any other tests (Table 2).

### 3.3 *Mtb* clearance during treatment

After one month of treatment, the bacterial detection rates in the definite TBM group were 3/38 (7.9%) by MBLA, 4/38 (10.5%) by MGIT, 19/38 (50.0%) by Xpert and 18/38 (47.4%) by ZN smear. The four patients’ positive by MGIT and three positive by MBLA did not overlap with each other and all were positive with Xpert at a low or very low level of detection (Ct value above 22). Of the four MGIT positive patients, one was isoniazid resistant, two died; and two of the three MBLA positive patients died.

**Figure 2:**
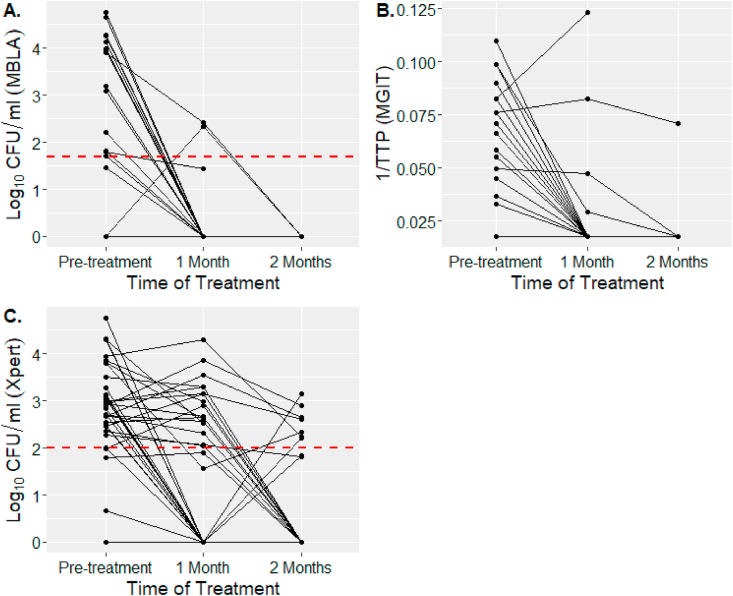
**Bacterial load change during 2 months of anti-TB treatment.** Bacteria load was measured in TBM patients who have follow-up CSF after 1 month (N = 38) and after 2 months (N = 32) by 3 methods: (**A**) MBLA (log_10_ CFU/ml), (**B**) MGIT culture (1/TTP) and (**C**) Xpert (log_10_ CFU/ml). Each black line in graphs represents one individual TBM patient. Red horizontal lines indicate in-vitro lower limit detection for MBLA (63 CFU/ml) or Xpert (100 CFU/ml). Ct cut-off was 35 for MBLA and 40 for Xpert. Samples above the Ct cut-offs were defined as 0 CFU/ml.

After two months of treatment, no cases 0/32 (0%) were positive by MBLA, only 1/32 (3.1%) by MGIT, 9/32 (28.1%) by Xpert and 5/32 (15.6%) by ZN smear (Table 3). Quantitative measurements of bacterial loads in CSF samples during treatment are shown in Fig. 2. After two months, almost all cases were negative and no cases reconverted from negative to positive between months one and two by these two methods (Fig. 2A and B). For Xpert, 50% of cases were detected as positive with bacterial loads 2–4 log_10_ CFU/ml after one month and 28.1% positive with bacterial loads 2–3 log_10_ CFU/ml after two months. Three cases reconverted from negative to positive between months one and two by Xpert while remaining negative by MGIT culture and survived after 9 months of treatment (Fig. 2C).

**Table 3:** *Mtb* detection rates by MBLA, MGIT, Xpert and ZN smear testing in pre-treatment, after 1 month and after 2 months CSF from all TBM patients.

Methods	Pre-treatment	After 1 month	After 2 months
	All TBM	TBM	TBM
	N = 99	N = 38	N = 32
**MBLA**	34 (34.3%)	3 (7.9%)	0 (0%)
**MGIT**	47 (47.5%)	4 (10.5%)	1 (3.1%)
**Xpert**	51 (51.5%)	19 (50%)	9 (28.1%)
**ZN smear**	55 (55.5%)	18 (47.4%)	5 (15.6%)

Data are number (%) of positive cases by methods.

Abbreviations: TBM, tuberculosis meningitis; MBLA; molecular bacterial load assay, MGIT; mycobacterial culture by MGIT (Becton Dickinson, USA), Xpert; Xpert MTB/RIF (Cepheid), ZN smear; Ziehl–Neelsen smear.

### 3.4 Detection limits of MBLA in CSF and sputum

The bacterial detection rate in pre-treatment CSF samples by MBLA (34.3%) was significantly lower than by the gold standard MGIT culture (47.5%). The reason could be that clinical samples contain potential inhibitors of RT-qPCR [17]. We hypothesized that 16S rRNA Ct values of MBLA were delayed in clinical samples compared to the GTC control. However, analysis shows that average Ct values of MBLA in CSF or in sputum were not significantly different from paired Ct values in the control for both low and high levels of BCG (Mean difference −0.46, 95% CI [-0.15; 1.07], *P* = 0.113 for CSF and 0.45, 95% CI [-0.09; 0.10], *P* = 0.088 for sputum). Lower limits of *Mtb* detection by MBLA were identified by serially diluting BCG in the GTC buffer, *Mtb*-negative sputum and CSF samples. The lowest concentrations of *Mtb* that could be detected and clearly distinguished from noise were 100 CFU/ml for sputum and 63 CFU/ml for CSF (Figure S1).

### 3.5 Bacterial load in CSF versus sputum samples

**Figure 3:**
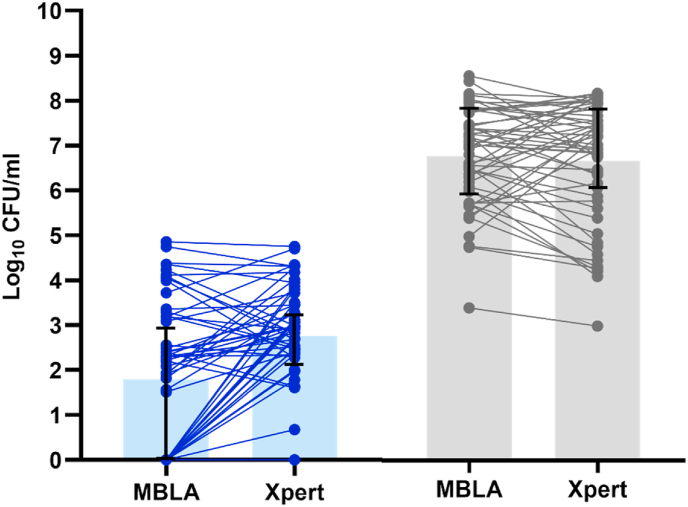
**Comparisons of pre-treatment bacterial load between TBM and PTB**. Bacteria load (log_10_ CFU/ml) was measured in pre-treatment CSF of 56 definite TBM patients (blue) and pre-treatment sputum of 56 PTB patients (grey) by MBLA and Xpert. Each horizontal line links bacterial load by MBLA with Xpert in the same individual. Median values (shaded bars) and 75% interquartile ranges (vertical lines) are shown. Ct cut-off was 35 for MBLA and 40 for Xpert. Samples above the Ct cut-offs were defined as 0 CFU/ml.

Bacterial load in CSF from TBM patients is known to be much lower than in sputum samples from PTB patients, making TBM difficult to diagnose and bacterial load hard to follow-up during treatment. In our previous study, median pre-treatment bacterial load from 56 AFB positive PTB patients was 7.02 [IQR 5.77; 7.78] log_10_ CFU/ml sputum by MBLA and 6.99 [IQR 6.03; 7.74] log_10_ CFU/ml sputum by Xpert [9]. In this study, median pre-treatment bacterial load from 56 definite-TBM patients was 2.09 [IQR 0.00; 3.19] log_10_ CFU/ml CSF by MBLA and 2.95 [IQR 2.35; 3.50] log_10_ CFU/ml CSF by Xpert. From these data, pre-treatment bacterial load in TBM samples was over ten thousand times (4–5 log_10_ fold) lower than in PTB samples (Fig. 3). In sputum, bacteria were detectable in all samples by both methods. In CSF, the 22/56 samples in which bacterial load was undetectable by MBLA mainly had low bacterial loads by Xpert, confirming that it is the low bacterial load which impedes the sensitivity of MBLA for detecting TBM.

## 4 Discussion

Deaths from TBM are prevented by the effective killing of *Mtb* by anti-tuberculosis drugs and the control of the intracerebral inflammatory response [18]. However, other than following the neurological status of the patient, primarily through the Glasgow Coma score, there are no validated laboratory methods to assess treatment response and bacterial killing, in particular. MBLA has been used in pulmonary TB to assess bacterial killing and the efficacy of anti-tuberculosis drugs early in treatment, but it has not yet been evaluated in TBM. We found MBLA, conducted on archived CSF, was less sensitive than MGIT culture and other routine methods in detecting *Mtb* before the start of treatment and the proportion of patients with a positive CSF MBLA after one month of treatment was very low (3/38).

Before treatment, MBLA showed a lower *Mtb* detection rate in TBM than MGIT culture, which is the traditional gold standard, and other routine microbiological tests (Xpert and ZN smear). ZN smear (55/99) was found to be superior to all other tests, which is consistent with our previous studies [5,15,19]. Reasons contributing to the high sensitivity of ZN smear could be optimized performance and a skillful microscopist in our laboratory, and non-culturable *Mtb* in MGIT culture. The ZN smear results were recorded only as positive or negative, so we could not compare MBLA results with smear grading. All 34 cases detected by MBLA were also positive by Xpert, a real-time PCR test that not only relatively quantifies bacterial level, but also predicts rifampicin resistance. Xpert (Ultra) has already been used to measure bacterial load in CSF from TBM patients in order to predict outcomes. High pre-treatment CSF *Mtb* load by Xpert in 692 Vietnamese adults with TBM was found predictive of new neurological events during treatment (p = 0.005), but not death [6]. Using Xpert Ultra in 102 Ugandans with HIV-associated TBM, pre-treatment CSF bacterial load was found to predict 2-week mortality, with high bacterial load in the low Ct value tertile associated with increased 2-week death (p = 0.01) [20]. Xpert (Ultra) has higher sensitivity in measuring pre-treatment bacterial load than MBLA, showing possible usefulness to identify patients at increased risk of neurological events or death. Pre-treatment, MBLA offers no diagnostic advantage if Xpert (Ultra) has already been performed.

After one and two months of treatment, the detection rates by both MGIT culture and MBLA were lower than Xpert and ZN smear. The MBLA results mirrored those of MGIT, suggesting they both detected viable, culturable bacteria during treatment. These results are similar to our findings in sputum samples from pulmonary TB patients [9]. The persisting positivity of Xpert and ZN stain suggest these tests detected both live and dead bacteria in the CSF and thus are not suitable for treatment monitoring [9]. Interestingly, the small numbers of patients who were persistently positive at 1 month by MBLA and/or MGIT had very poor outcomes, suggesting the detection of viable bacteria in CSF may have prognostic significance. Larger studies are required to confirm or refute this finding.

Death or neurological disability still occurs in half of all TBM cases and around 90% of deaths occur during the first three months of treatment, so it might be important to monitor treatment response with viable bacterial numbers at very early time points (after 1–2 weeks of treatment, for example). Viable bacterial detection in definite TBM cases by MBLA and MGIT declined rapidly after one month (7.9% and 10.5%) and two months (0% and 3.1%) treatment. This suggests that monthly monitoring of bacterial load in TBM might be too infrequent and earlier time-points might provide more useful information. However, the possibility of gaining more information from more regular lumbar punctures and CSF sampling needs to be balanced against the discomfort and small but significant risk of procedure-associated complications.

It is unclear why the pre-treatment *Mtb* detection rate in CSF by MBLA was significantly lower than for other methods and lower than in other studies. Previous studies have suggested that the low sensitivity of PCR-based tests to detect bacteria in CSF may be caused by high levels of glucose, proteins, red blood cell contamination and co-extraction of human genomic materials disrupting the performance of the assay [17,21]. However, our data showed MBLA Ct values in both CSF and sputum samples spiked with low and high levels of BCG were not significantly different from Ct values in GTC controls. This indicates that the inhibition in CSF, which was reported for other PCR-based tests, did not affect the performance of MBLA in our study. An explanation for the absence of inhibition could be the high efficiency of the RNA extraction method in MBLA. The extraction uses a high concentration of strong chaotropic guanidine thiocyanate which can effectively denature proteins and lyse human cells, so helps remove inhibitors in clinical samples before bacterial RNA is extracted. RNA extraction also involves a homogenization step at high speed which may disrupt the tough bacterial cell wall completely to maximize RNA recovery [17].

Using a combined 10-fold and 2-fold serial dilution, we found that the LOD of *Mtb* in CSF by MBLA was 63 CFU/ml. This value is similar to previously reported values of 100 CFU/ml [13] or 84 CFU/ml [22]. Xpert and MGIT culture, currently routinely used in diagnosis of TBM, are reported to have LODs of around 114–131 CFU/ml and 10–100 CFU/ml respectively [23]. Since MBLA has shown no inhibition and comparative LOD with other routine tests, the low detection rate of MBLA in CSF may due to the multiple centrifugation steps in RNA extraction that have already been shown to result in loss of RNA, affecting the test sensitivity (Sabiiti, personal communication). An automated MBLA system with integrated RNA extraction and amplification for RT-qPCR as in Xpert might increase RNA yield and improve detection. During treatment, if as sensitive as culture, MBLA would be preferable to culture due to its faster turnaround time. While culture is useful for research purposes, it is too slow to be of clinical use in monitoring treatment response. Directly comparing CSF versus sputum samples, the pre-treatment bacterial load in CSF were 4–5 log_10_ fold lower than in sputum, and therefore the loads drop below the MBLA LOD during treatment. In both types of sample, the values have a wide range but there is almost no overlap. This big difference makes TBM more difficult to diagnose and treatment response much harder to monitor.

In summary, serial CSF MGIT and MBLA may help monitor treatment response in TBM. ZN smear and Xpert remain positive for much longer after the start of treatment than MBLA and MGIT, and the prognostic significance of positive results are uncertain. Those persistently positive by MBLA or MGIT had poor outcomes, although numbers were too small to be definitive, suggesting further studies would be informative. The reason for the reduced sensitivity of MBLA, especially before the start of treatment, was surprising and of uncertain cause. Previous studies have reported MBLA was more sensitive than both MGIT and smear [9,11]. It is possible that the processing and long-term storage of the samples tested in this study may have reduced the performance of MBLA. In addition, CSF sampling earlier in the first month of TBM treatment may provide more informative data. Thus further prospective studies, testing fresh CSF early in treatment and linked to careful assessment of clinical outcomes, are required to judge definitively the value of MBLA in TBM treatment monitoring.

## Author contributions statement

H.T.H. and N.T.T.T designed the research; H.T.H. and D.D.A.T performed tuberculosis diagnostic tests and experiments; H.T.H. and N.T.T.T analysed data; N.H.P and G.E.T led the clinical trial, collected samples and clinical data; W.S. and S.H.G. provided BCG and internal control strains; H.T.H., N.T.T.T, G.E.T, W.S. and S.H.G wrote the article.

## Declaration of competing interest

The authors declare that the research was conducted in the absence of any commercial or financial relationships that could be construed as a potential conflict of interest.

## Appendix A Supplementary data

The following is the supplementary data related to this article:Multimedia component 1 Supplement Figure-S1. Performance of MBLA on clinical CSF and sputum samples compared to GTC control at a range of 1 log_10_ to 7 log_10_ CFU/ml BCG bacterial load. BCG strain was cultured and quantified by Miles and Misra method (The Journal of Hygiene. 1938; 38(6):732-49). Stored BCG from culture was 10 fold serially diluted and spiked at concentrations of 1 log 10 to 7 log_10_ CFU/ml into (A) GTC buffer control, (B) pooled sputum. In (C) pooled CSF, stored BCG was 10 fold serially diluted and spiked from 3 log_10_ to 7 log_10_ CFU/ml then 2 fold serially diluted and spiked from 1 log_10_ to 3 log_10_ CFU/ml into CSF. MBLA was performed and 16S rRNA Ct values recorded. Linear regression line represents the association between Ct values of MBLA and BCG concentration. Error bars represent mean and standard deviation of BCG concentration from 4 replicates. Multimedia component 1
