# Supplementary figures and images for: Evaluation of the molecular bacterial load assay for detecting viable Mycobacterium tuberculosis in cerebrospinal fluid before and during tuberculous meningitis treatment
Source: Tuberculosis (Edinb). 2021 May;128:102084. doi: 10.1016/j.tube.2021.102084 (PMC8204225; doi:10.1016/j.tube.2021.102084)

**Supplement Figure S1:**


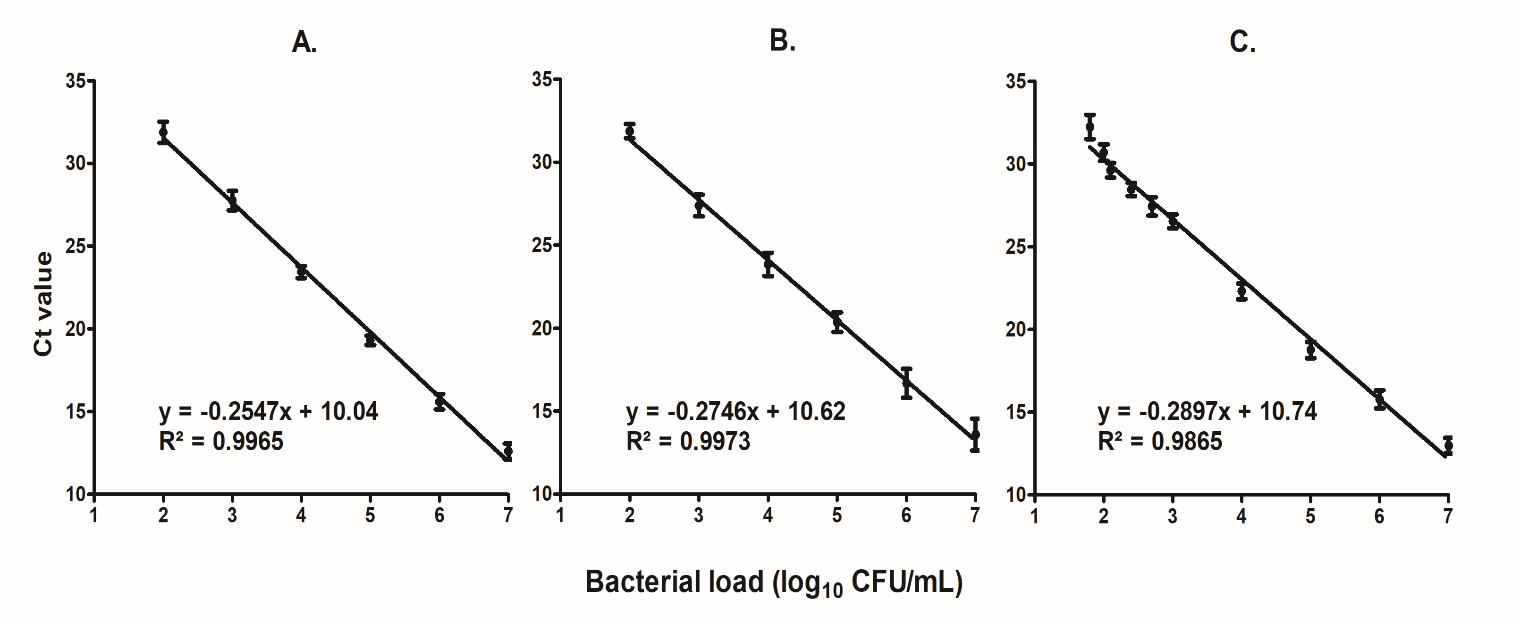

Supplement: The following is the supplementary data related to this article:Multimedia component 1 — Supplement Figure-S1. Performance of MBLA on clinical CSF and sputum samples compared to GTC control at a range of 1 log10 to 7 log10 CFU/ml BCG bacterial load. BCG strain was cultured and quantified by Miles and Misra method (The Journal of Hygiene. 1938; 38(6):732-49). Stored BCG from culture was 10 fold serially diluted and spiked at concentrations of 1 log 10 to 7 log10 CFU/ml into (A) GTC buffer control, (B) pooled sputum. In (C) pooled CSF, stored BCG was 10 fold serially diluted and spiked from 3 log10 to 7 log10 CFU/ml then 2 fold serially diluted and spiked from 1 log10 to 3 log10 CFU/ml into CSF. MBLA was performed and 16S rRNA Ct values recorded. Linear regression line represents the association between Ct values of MBLA and BCG concentration. Error bars represent mean and standard deviation of BCG concentration from 4 replicates. [file mmc1.docx]
